# Exercise Hemodynamics and Quality of Life after Aortic Valve Replacement for Aortic Stenosis in the Elderly Using the Hancock II Bioprosthesis

**DOI:** 10.1155/2014/151282

**Published:** 2014-12-02

**Authors:** Theodore Long, Becky M. Lopez, Christopher Berberian, Mark J. Cunningham, Vaughn A. Starnes, Robbin G. Cohen

**Affiliations:** ^1^Robert Wood Johnson Clinical Scholars Program, Department of Internal Medicine, Yale School of Medicine, New Haven, CT 06520, USA; ^2^Department of Cardiothoracic Surgery, Keck School of Medicine, University of Southern California, Los Angeles, CA 90033, USA; ^3^USC Healthcare Consultation Center II, 1520 San Pablo Street, Suite 4300, Los Angeles, CA 90033, USA

## Abstract

*Background and Aim*. While aortic valve replacement for aortic stenosis can be performed safely in elderly patients, there is a need for hemodynamic and quality of life evaluation to determine the value of aortic valve replacement in older patients who may have age-related activity limitation. *Materials and Methods*. We conducted a prospective evaluation of patients who underwent aortic valve replacement for aortic stenosis with the Hancock II porcine bioprosthesis. All patients underwent transthoracic echocardiography (TTE) and completed the RAND 36-Item Health Survey (SF-36) preoperatively and six months postoperatively. *Results*. From 2004 to 2007, 33 patients were enrolled with an average age of 75.3 ± 5.3 years (24 men and 9 women). Preoperatively, 27/33 (82%) were New York Heart Association (NYHA) Functional Classification 3, and postoperatively 27/33 (82%) were NYHA Functional Classification 1. Patients had a mean predicted maximum *V*_O_2__ (mL/kg/min) of 19.5 ± 4.3 and an actual max *V*_O_2__ of 15.5 ± 3.9, which was 80% of the predicted *V*_O_2__. Patients were found to have significant improvements (*P* ≤ 0.01) in six of the nine SF-36 health parameters. *Conclusions*. In our sample of elderly patients with aortic stenosis, replacing the aortic valve with a Hancock II bioprosthesis resulted in improved hemodynamics and quality of life.

## 1. Introduction

Aortic valve replacement (AVR) in elderly patients can be performed with excellent results and can improve survival when compared with medical therapy for patients with aortic stenosis [1–3]. However, little is known regarding postoperative recovery and subsequent quality of life (QOL) in patients who undergo either isolated AVR or AVR associated with other procedures such as coronary artery bypass grafting (CABG). For instance, controversy continues to exist regarding the value of aortic valve replacement in older patients whose lifestyles and activities may be limited by their ages or comorbidities. We used echocardiography (ECHO), bicycle cardiopulmonary exercise testing (CPET), and QOL surveys to evaluate elderly patients after AVR for aortic stenosis with the Hancock II porcine bioprosthesis (Medtronic Inc., Minneapolis, MN).

## 2. Methods

This prospective study was approved by the institutional review board, and written informed consent was obtained from all study participants. All patients underwent transthoracic echocardiography (TTE) and completed the RAND 36-Item Health Survey (SF-36) as part of their preoperative evaluation. Six months later they underwent repeat TTE combined with CPET and completed a second SF-36.

### 2.1. Aortic Valve Replacement

All patients underwent open aortic valve replacement using standard cardiopulmonary bypass techniques and the Hancock II porcine bioprosthesis (Medtronic Inc.). After complete debridement of the aortic annulus via a transverse aortotomy, manufacturer provided valve sizers were used to select the valve size that most closely approximated the aortic outflow orifice while sitting comfortably in the supra-annular position. The valve was then implanted using synthetic sutures reinforced with teflon felt pledgets on the ventricular side. Transesophageal echocardiography was utilized after weaning from cardiopulmonary bypass to assure adequate valve function and to assess for perivalvular leaks. Postoperative anticoagulation was limited to ASA only for patients in sinus rhythm.

### 2.2. CPET Protocol with Bicycle Ergometer

Peak exercise capacity is defined as the “maximum ability of the cardiovascular system to deliver oxygen to exercising skeletal muscle, and of the exercising muscle to extract oxygen from the blood [4].” Exercise capacity is determined by (1) pulmonary gas exchange, (2) cardiovascular performance, (3) and skeletal muscle metabolism. Prior to recording data, a clamp was placed on the nose and the patient breathed through a mouthpiece attached to a nonrebreathing valve. Expired air was collected through the valve into a mixing chamber and sampled continuously by fast responding oxygen and carbon dioxide analyzers. This method uses a distributive processing technique involving a waveform analyzer and a host computer. Respiratory gas exchange was obtained on a breath by breath analysis.

CPET was conducted on an upright bicycle ergometer utilizing a ramp protocol with increments of 10–15 watts per minute [5]. Baseline measurements were established during three minutes of rest and three minutes of unloaded cycling. Testing was ended when *V*_O_2__ *Max*⁡, the maximum capacity of an individual during incremental exercise to transport and utilize oxygen, was obtained. Breath analysis was used to measure respiratory gas exchange through obtaining *V*_O_2__ and central venous oxygen saturation (CVO_2_). Ventilation, the rate that air is exchanged between the lungs and the environment, was also measured through breath analysis. Peak *V*_O_2__ was determined and the minute ventilation-carbon dioxide production (VE/CVO_2_) slope was used to estimate the anaerobic threshold (AT).

Workload and max heart rate (HR) and blood pressure (BP) were also measured. AT, the point during exercise where muscles derive energy from nonoxygenic sources, of <40% of the predicted peak *V*_O_2__ was considered pathologically reduced and indicative of circulatory insufficiency. A respiratory exchange ratio, the ratio of carbon dioxide to percent oxygen in a breath, of <1.0 in the absence of other metabolic abnormalities suggested poor effort or anxiety, because the ratio of carbon dioxide is expected to increase with exercise. Determination of peak *V*_O_2__ was derived as the average of two consecutive 15- or 20-second segments from the last minute of exercise with the highest average value. CPET results for each patient were compared with those from predicted normal patients of a similar age as described by Fleg and Lakatta [6].

### 2.3. Doppler ECHO

Color flow Doppler ECHO of the prosthetic aortic valve in each patient was performed at rest and immediately after peak *V*_O_2__ was reached with CPET. Mean and peak gradients were measured. We also measured left ventricular function via ejection fraction on ECHO.

### 2.4. Quality of Life

Patients completed the SF-36 survey pre- and postoperatively at six months. The SF-36 is a short-form health survey with eight sections describing functional health and well-being. It includes psychometrically based physical and mental health scores. The SF-36 is a generic tool that can be used for any population or age group. We used matched pairs *t*-tests to compare preoperative to postoperative SF-36 scores in order to assess improvement in QOL [7, 8].

## 3. Results

Thirty three patients underwent aortic valve replacement with a porcine Hancock II bioprosthesis. The average age was 75.3 ± 5.3 years (24 men and 9 women). NYHA functional class pre- and postoperatively is shown in Table 1. Twenty-three concomitant procedures were completed as follows: 12 CABG, three ascending aorta replacements, two mitral valve repairs, two mazes, one ASD closure, one carotid endarterectomy, one PFO closure, and one SVC enlargement.

### 3.1. Hemodynamic Results

**Figure 1:**
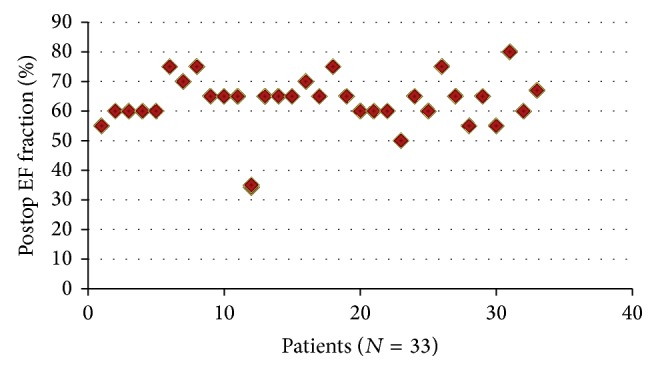
Left ventricular function.

Mean pressure gradient at rest and during exercise and BSA are depicted in Table 2 according to valve size.  Figure 1 displays the postoperative ejection fractions for our patients.

### 3.2. Bicycle CPET

**Figure 2:**
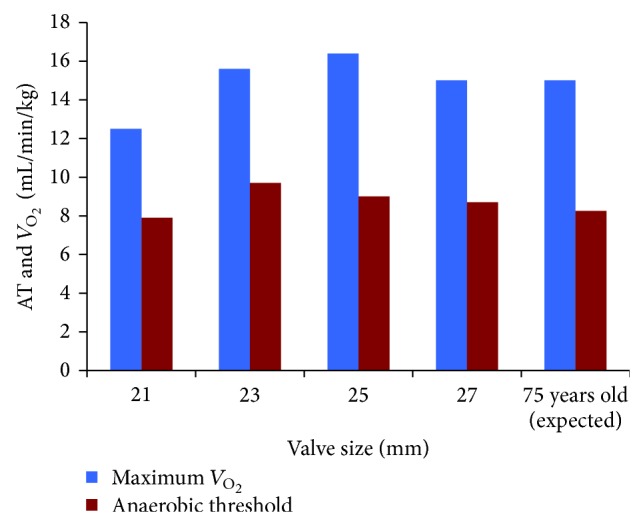
Max *V*_O_2__ and anaerobic threshold.

Patients had a mean predicted maximum *V*_O_2__ (mL/kg/min) of 19.5 ± 4.3 and an actual max *V*_O_2__ of 15.5 ± 3.9, which was 80% of the predicted *V*_O_2__. The expected maximum *V*_O_2__ used for a healthy 75-year-old person was 19 mL/kg/min predicted and 15 mL/kg/min actual, resulting in 80% of the predicted *V*_O_2__ for a healthy subject [6, 9]. The max *V*_O_2__ values for our patients are displayed in Figure 2. AT greater than 55% of the max *V*_O_2__ is thought to be a normal response [10, 11].

### 3.3. RAND SF-36 Survey Results

Patients showed statistically significant improvements (*P* ≤ 0.01) in six of nine health parameters outlined by the QOL survey. Results from the SF-36 survey are shown in Table 3.

## 4. Discussion

The Hancock II (Medtronic Inc.) is a second generation supra-annular porcine bioprosthesis with a proven history of excellent durability and acceptable hemodynamic performance [12]. Though third generation bioprostheses are currently available and have been designed with improved durability and hemodynamics in mind, they are significantly more expensive and their advantages remain controversial. While pericardial aortic bioprostheses are known to have improved hemodynamics over their porcine counterparts, this does not necessarily translate into improved survival or left ventricular mass regression [13]. Furthermore, long-term studies comparing the durability of third generation valves (with new anticalcification agents) with their second generation counterparts are not yet available. The result is that the Hancock II continues to see widespread use, both in the United States and other countries where price and availability may take on greater roles.

We evaluated aortic valve replacement with the Hancock II porcine bioprosthesis in elderly patients with aortic stenosis with regard to symptom improvement and quality of life, exercise tolerance, and prosthetic hemodynamic performance. Our study is one of the few that contributes to both subjective and objective assessment of the benefits of valve replacement in the elderly.

In vivo comparisons of bioprosthetic valves can be difficult, due to the clinical and hemodynamic variability that occur between measurements [14, 15]. Whereas one year seems to be the preferred followup for echocardiographic analysis of bioprosthetic hemodynamics, we chose six months because we were primarily interested in early recovery from aortic valve surgery with regard to exercise capacity and quality of life. We limited our echocardiographic analysis to mean pressure gradients and EOA, given their common use in surgical studies of this kind.

The exercise hemodynamics in our patients showed a lower mean pressure gradient by labeled valve size than patients with bioprosthetic valves in other studies. Eichinger et al. looked at the Medtronic Mosaic valve in 2005 and found that the mean pressure gradients at maximum stress (75 watts) were 25.1, 25.7 ± 5.6, and 20.2 ± 8.0 for valves sized 21 mm, 23 mm, and 25 mm, respectively [16]. Our results show a lower mean pressure gradient for the 23 mm valve (20.1 ± 8.1) and the 25 mm valve (20.1 ± 10.2). We found a higher mean pressure gradient for the 21 mm valve (27.2 ± 14.0) compared to 25.1 found by Eichinger. However, this result in the Eichinger study was a single patient. Overall, our results show similar mean pressure gradients to previous studies that evaluated porcine bioprostheses [16, 17]. We also found a decrease in pressure gradients at both rest and peak exercise with an increase in valve size, which is consistent with existing literature.

Our patients demonstrated similar predicted and actual maximum *V*_O_2__ measurements to the expected normal aging population [6, 9]. They were also found to have an AT greater than 55% of the maximum *V*_O_2__, which is consistent with a normal response [10, 11]. The fact that our patients had exercise testing results that were within normal limits for their age suggests that these patients had an excellent functional response to AVR.

To evaluate quality of life, we employed the SF-36 survey, which has been shown to have a high discriminatory power as well as test-retest reliability and construct validity [7, 8, 18]. We found that patients showed significant improvements (*P* ≤ 0.01) in QOL six months postoperatively in six of the nine SF-36 health parameters: physical functioning, role limitations due to health, energy-fatigue, social functioning, general health, and health change. This result suggests that the patients in our study had a significant decrease in symptoms and overall improvement in QOL.

Subjective assessment of QOL is an uncommonly used but important method of assessment of postsurgical evaluation. In fact, others have found that only 3.6% of cardiovascular randomized controlled trials reported QOL data, and when QOL results are reported, if they are discordant, authors will not look at QOL data in relation to the other study outcomes [19, 20]. Our QOL results show significant and meaningful improvements for patients after valve replacement. These results are consistent with results from other studies that have found QOL improvement in elderly patients with bioprosthetic heart valve replacement [21–23].

Our study had several limitations. First, our in vivo ECHO analysis was six months after surgery, as opposed to one year that has been used to evaluate bioprosthetic hemodynamics in other studies. However, we were interested in studying early recovery to compare with quality of life metrics. Second, while a midterm analysis with exercise capacity would be ideal in terms of long-term performance evaluation, this analysis was outside of the scope of our study.

We found that replacing a stenotic aortic valve with a Hancock II porcine bioprosthesis resulted in low mean pressure gradients during both rest and peak exercise, above average exercise capacity at six months postoperatively, and significant improvement in overall quality of life. Given the excellent durability recently reported with this valve, the Hancock II remains an excellent option for aortic valve replacement in elderly patients [3].

## Figures and Tables

**Table 1:** NYHA Functional Classification.

Functional Classification	Pre-op	Post-op
FC I	1 (3%)	27 (82%)
FC II	5 (15%)	6 (18%)
FC III	27 (82%)	—
FC IV	—	—

**Table 2:** Post-op ECHO results.

Valve size (mm)	Number of patients	Mean gradient at rest (mmHg)	Mean gradient at exercise (mmHg)	BSA (m^2^)
21	5	14.2 ± 5.5	27.2 ± 14.0	1.88 ± 0.15
23	13	12.4 ± 5.9	20.1 ± 8.1	2.01 ± 0.15
25	14	10.1 ± 3.7	20.1 ± 10.2	2.05 ± 0.17
27	1	12	17	1.8
All sizes	33	11.7 ± 5.0	21.1 ± 10.0	2.0 ± 0.17

**Table 3:** SF-36 QOL.

Health parameters	Items	Pre-op	Post-op	*P* value
Physical functioning	10	52 ± 27	73 ± 24	≤0.001
Role limitations due to physical health	4	32 ± 40	72 ± 36	≤0.001
Role limitations due to emotional health	3	61 ± 40	74 ± 38	0.096
Energy-fatigue	4	54 ± 22	68 ± 20	≤0.001
Emotional well-being	5	78 ± 16	81 ± 16	0.264
Social functioning	2	74 ± 28	88 ± 18	≤0.001
Pain	2	75 ± 28	82 ± 22	0.068
General health	5	66 ± 16	75 ± 13	0.01
Health change	1	32 ± 22	80 ± 23	≤0.001

## References

[1] Likosky D. S., Sorensen M. J., Dacey L. J. (2009). Long-term survival of the very elderly undergoing aortic valve surgery. *Circulation*.

[2] Varadarajan P., Kapoor N., Bansal R. C., Pai R. G. (2006). Survival in elderly patients with severe aortic stenosis is dramatically improved by aortic valve replacement: results from a cohort of 277 patients aged ≥80 years. *European Journal of Cardio-Thoracic Surgery*.

[3] David T. E., Armstrong S., Maganti M. (2010). Hancock II bioprosthesis for aortic valve replacement: the gold standard of bioprosthetic valves durability?. *Annals of Thoracic Surgery*.

[4] Braunwald E. (1992). *Heart Disease: A Textbook of Cardiovascular Medicine*.

[5] Lear S. A., Brozic A., Myers J. N., Ignaszewski A. (1999). Exercise stress testing: an overview of current guidelines. *Sports Medicine*.

[6] Fleg J. L., Lakatta E. G. (1988). Role of muscle loss in the age-associated reduction in VO2 max. *Journal of Applied Physiology*.

[7] Brazier J. E., Harper R., Jones N. M. B., O'Cathain A., Thomas K. J., Usherwood T., Westlake L. (1992). Validating the SF-36 health survey questionnaire: new outcome measure for primary care. *British Medical Journal*.

[8] Ware J. E., Kosinski M., Bayliss M. S., McHorney C. A., Rogers W. H., Raczek A. (1995). Comparison of methods for the scoring and statistical analysis of SF-36 health profile and summary measures: summary of results from the Medical Outcomes Study. *Medical Care*.

[9] Arena R., Myers J., Williams M. A., Gulati M., Kligfield P., Balady G. J., Collins E., Fletcher G. (2007). Assessment of functional capacity in clinical and research settings: a scientific statement from the American Heart Association committee on exercise, rehabilitation, and prevention of the council on clinical cardiology and the council on cardiovascular nursing. *Circulation*.

[10] Matsumura N., Nishijima H., Kojima S., Hashimoto F., Minami M., Yasuda H. (1983). Determination of anaerobic threshold for assessment of functional state in patients with chronic heart failure. *Circulation*.

[11] Wasserman K., Whipp B. J., Koyal S. N., Beaver W. L. (1973). Anaerobic threshold and respiratory gas exchange during exercise. *Journal of Applied Physiology*.

[12] Valfrè C., Ius P., Minniti G. (2010). The fate of Hancock II porcine valve recipients 25 years after implant. *European Journal of Cardio-Thoracic Surgery*.

[13] Chan V., Kulik A., Tran A., Hendry P., Masters R., Mesana T. G., Ruel M. (2010). Long-term clinical and hemodynamic performance of the hancock II versus the perimount aortic bioprostheses. *Circulation*.

[14] Gerosa G., Tarzia V., Rizzoli G., Bottio T. (2006). Small aortic annulus: the hydrodynamic performances of 5 commercially available tissue valves. *The Journal of Thoracic and Cardiovascular Surgery*.

[15] Bottio T., Tarzia V., Rizzoli G., Gerosa G. (2008). The changing spectrum of bioprostheses hydrodynamic performance: considerations on in-vitro tests. *Interactive Cardiovascular and Thoracic Surgery*.

[16] Eichinger W. B., Botzenhardt F., Keithahn A., Guenzinger R., Bleiziffer S., Wagner I., Bauernschmitt R., Lange R. (2005). Exercise hemodynamics of bovine versus porcine bioprostheses: a prospective randomized comparison of the Mosaic and Perimount aortic valves. *The Journal of Thoracic and Cardiovascular Surgery*.

[17] Chambers J. B., Rajani R., Parkin D., Rimington H. M., Blauth C. I., Venn G. E., Young C. P., Roxburgh J. C. (2008). Bovine pericardial versus porcine stented replacement aortic valves: early results of a randomized comparison of the Perimount and the Mosaic valves. *The Journal of Thoracic and Cardiovascular Surgery*.

[18] Shephard R. J., Franklin B. (2001). Changes in the quality of life: a major goal of cardiac rehabilitation. *Journal of Cardiopulmonary Rehabilitation*.

[19] Contopoulos-Ioannidis D. G., Karvouni A., Kouri I., Ioannidis J. P. A. (2009). Reporting and interpretation of SF-36 outcomes in randomised trials: systematic review. *BMJ*.

[20] Sanders C., Egger M., Donovan J., Tallon D., Frankel S. (1998). Reporting on quality of life in randomised controlled trials: bibliographic study. *British Medical Journal*.

[21] Goldsmith I. R., Lip G. Y., Patel R. L. (2001). A prospective study of changes in patients' quality of life after aortic valve replacement. *The Journal of Heart Valve Disease*.

[22] Sedrakyan A., Hebert P., Vaccarino V., Paltiel A. D., Elefteriades J. A., Mattera J., Lin Z., Roumanis S. A., Krumholz H. M. (2004). Quality of life after aortic valve replacement with tissue and mechanical implants. *The Journal of Thoracic and Cardiovascular Surgery*.

[23] Taillefer M.-C., Dupuis G., Hardy J.-F., LeMay S. (2005). Quality of life before and after heart valve surgery is influenced by gender and type of valve. *Quality of Life Research*.

